# Supplementary material for: Identification of Unstable Ellagitannin Metabolites in the Leaves of Quercus dentata by Chemical Derivatization
Source: Molecules. 2023 Jan 27;28(3):1246. doi: 10.3390/molecules28031246 (PMC9919081; doi:10.3390/molecules28031246)

Supplementary Materials

Chemical evidence for a missing intermediate in oxidative degradation of oak ellagitannin vescalagin

Zhangbin Liu [1,2], Yosuke Matsuo [1], Yoshinori Saito [1], Yong-Lin Huang [2], Dian-Peng Li [2], and Takashi Tanaka [1,*]

1 Department of Natural Product Chemistry, Graduate School of Biomedical Sciences, Nagasaki University, 1-14 Bunkyo-machi, Nagasaki 852-8521, Japan

2 Guangxi Key Laboratory of Functional Phytochemicals Research and Utilization, Guangxi Institute of Botany, Guilin 541006, China

* Correspondence: t-tanaka@nagasaki-u.ac.jp; Tel.: +81-95-819-2432 (T.T.)

Figure S1. ^{1}H NMR spectrum of **6** in acetone-d_6.

Figure S2. ^{13}C NMR spectrum of **6** in acetone-d_6.

Figure S3. ^{1}H-^{1}H COSY spectrum of **6** in acetone-d_6.

Figure S4. HSQC spectrum of **6** in acetone-d_6.

Figure S5. HMBC spectrum of **6** in acetone-d_6.

Figure S6. Roesy spectrum of **6** in acetone-d_6.

Figure S7. HR-FAB-MS spectrum of **6**.

Figure S8. ^{1}H NMR spectrum of **7** in acetone-d_6.

Figure S9. ^{13}C NMR spectrum of **7** in acetone-d_6.

Figure S10. ^{1}H-^{1}H COSY spectrum of **7** in acetone-d_6.

Figure S11. HSQC spectrum of **7** in acetone-d_6.

Figure S12. HMBC spectrum of **7** in acetone-d_6 (measured at 10Hz).

Figure S13. HMBC spectrum of **7** in acetone-d_6 (measured at 5Hz).

Figure S14. NOESY spectrum of **7**.

Figure S15. HR-FAB-MS spectrum of **7**.

Figure S16. ^{1}H NMR spectrum of **8** in acetone-d_6.

Figure S17. ^{13}C NMR spectrum of **8** in acetone-d_6.

Figure S18. ^{1}H-^{1}H COSY spectrum of **8** in acetone-d_6.

Figure S19. HSQC spectrum of **8** in acetone-d_6.

Figure S20. HMBC spectrum of **8** in acetone-d_6.

Figure S21. NOESY spectrum of **8**.

Figure S22. FAB-MS spectrum of **8.**

Figure S1. ^{1}H NMR spectrum of **6** in acetone-d_6.

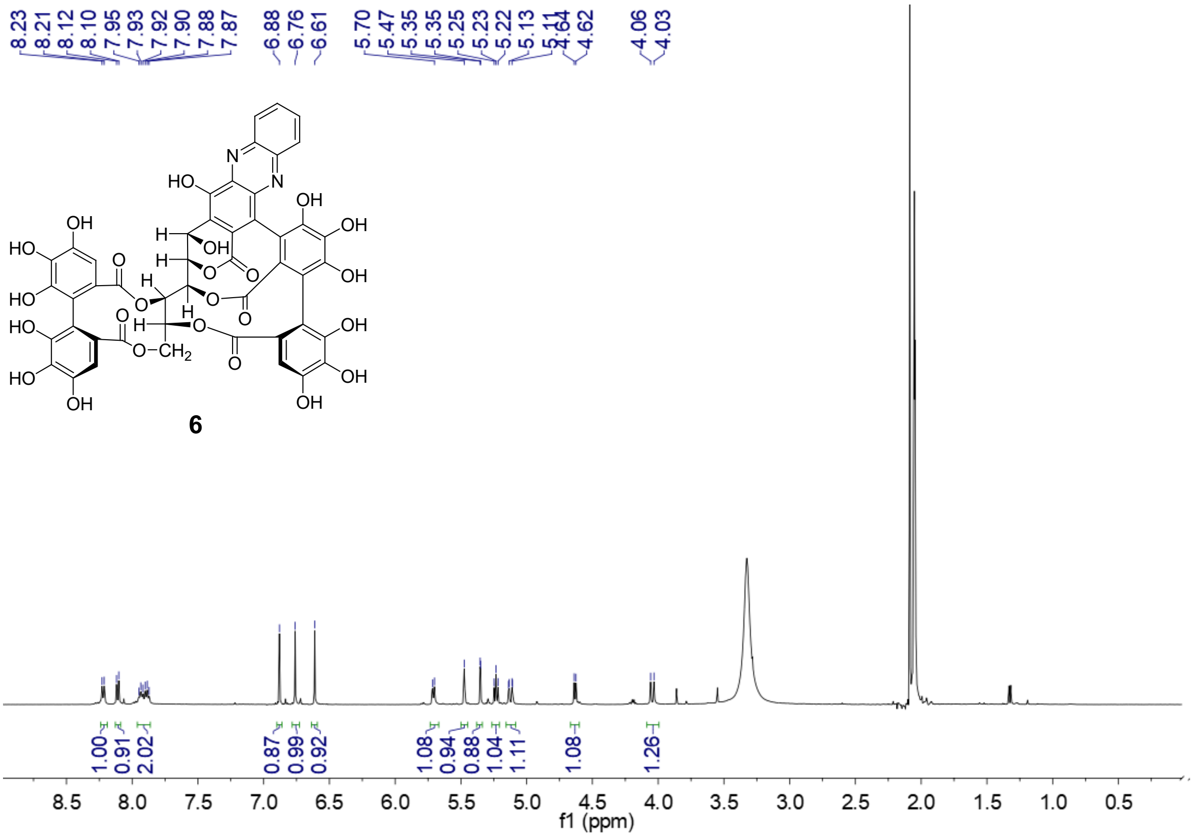

Figure S2. ^{13}C NMR spectrum of **6** in acetone-d_6.

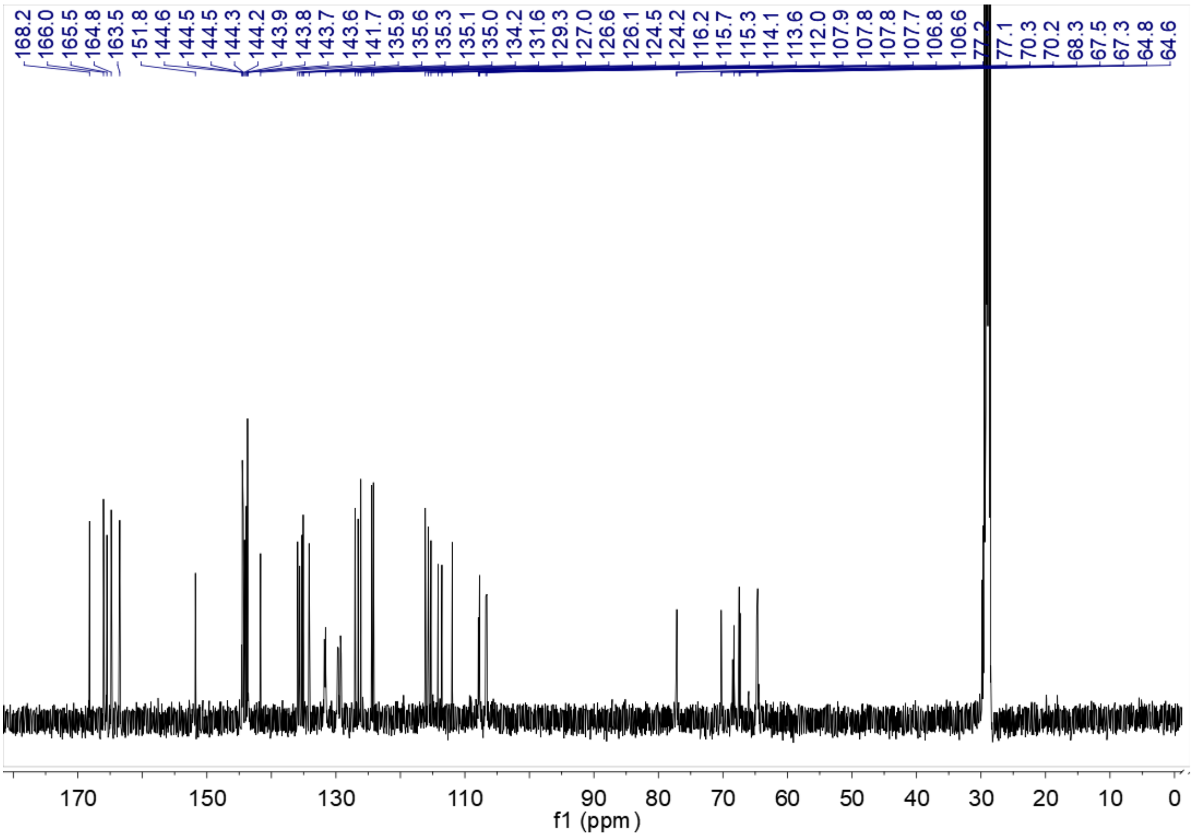

Figure S3. ^{1}H-^{1}H COSY spectrum of **6** in acetone-*d*6.

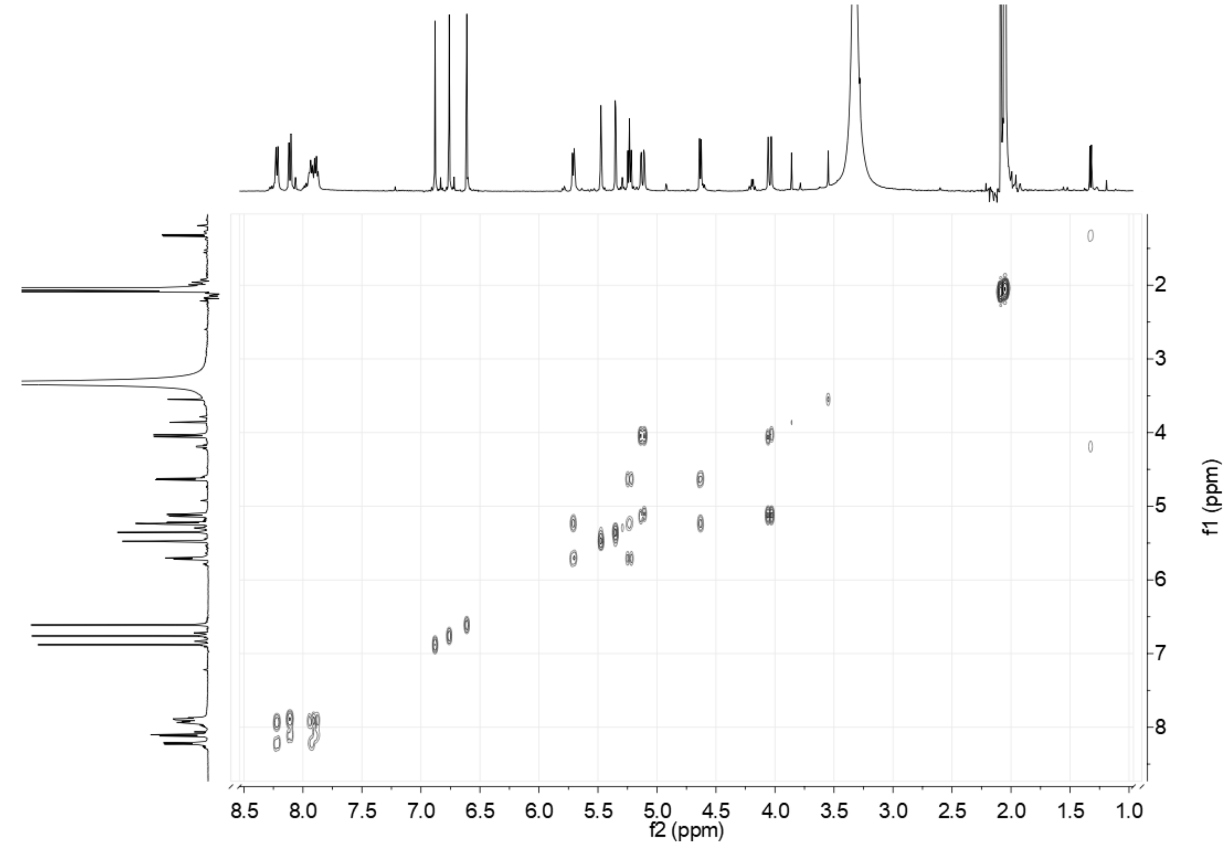

Figure S4. HSQC spectrum of **6** in acetone-*d*6.

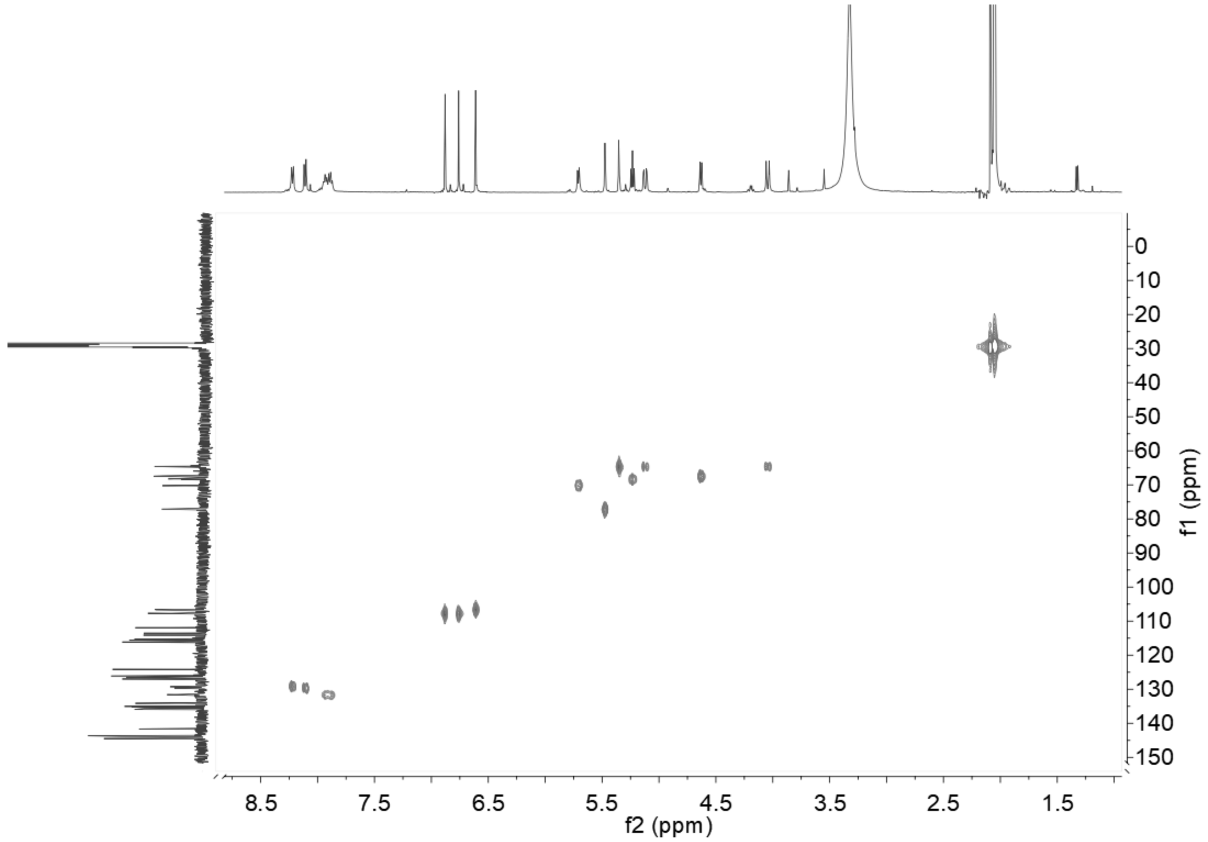

Figure S5. HMBC spectrum of **6** in acetone-d_6.

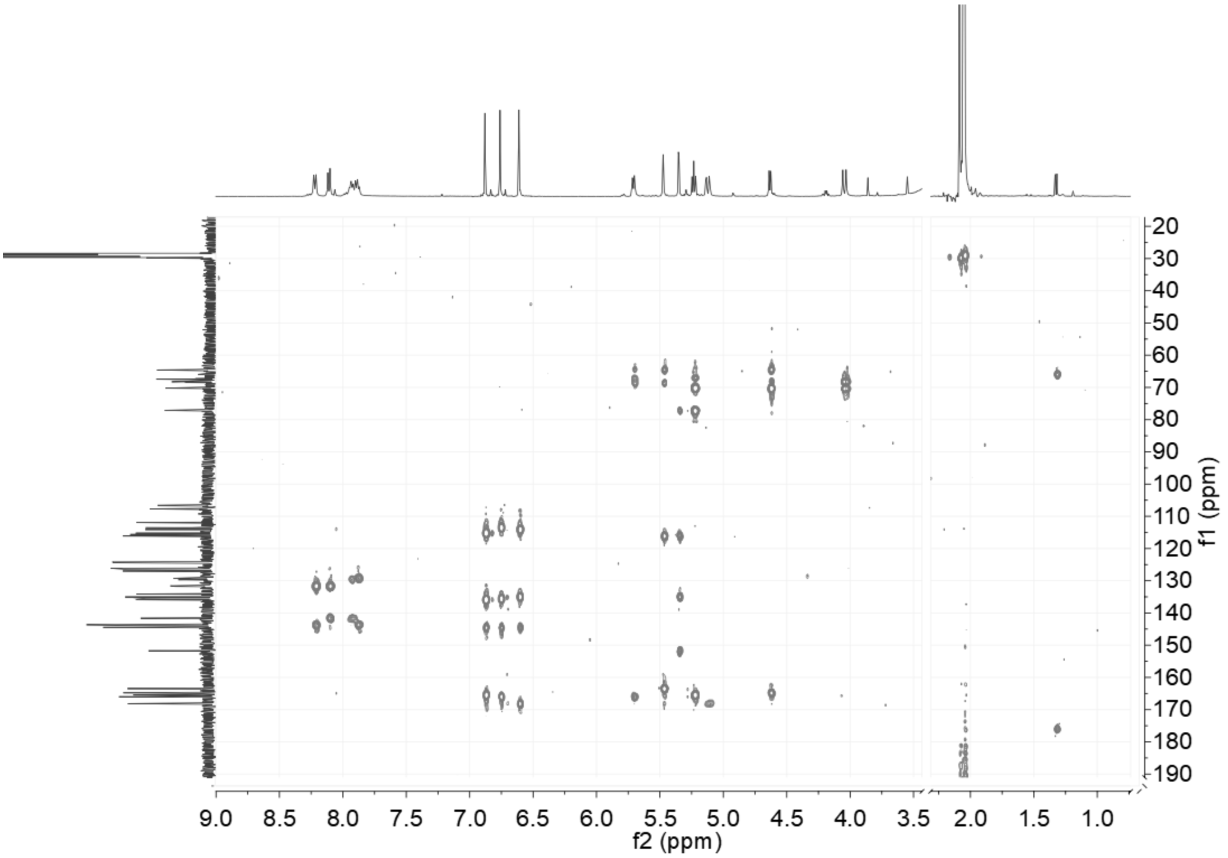

Figure S6. Roesy spectrum of **6** in acetone-d_6.

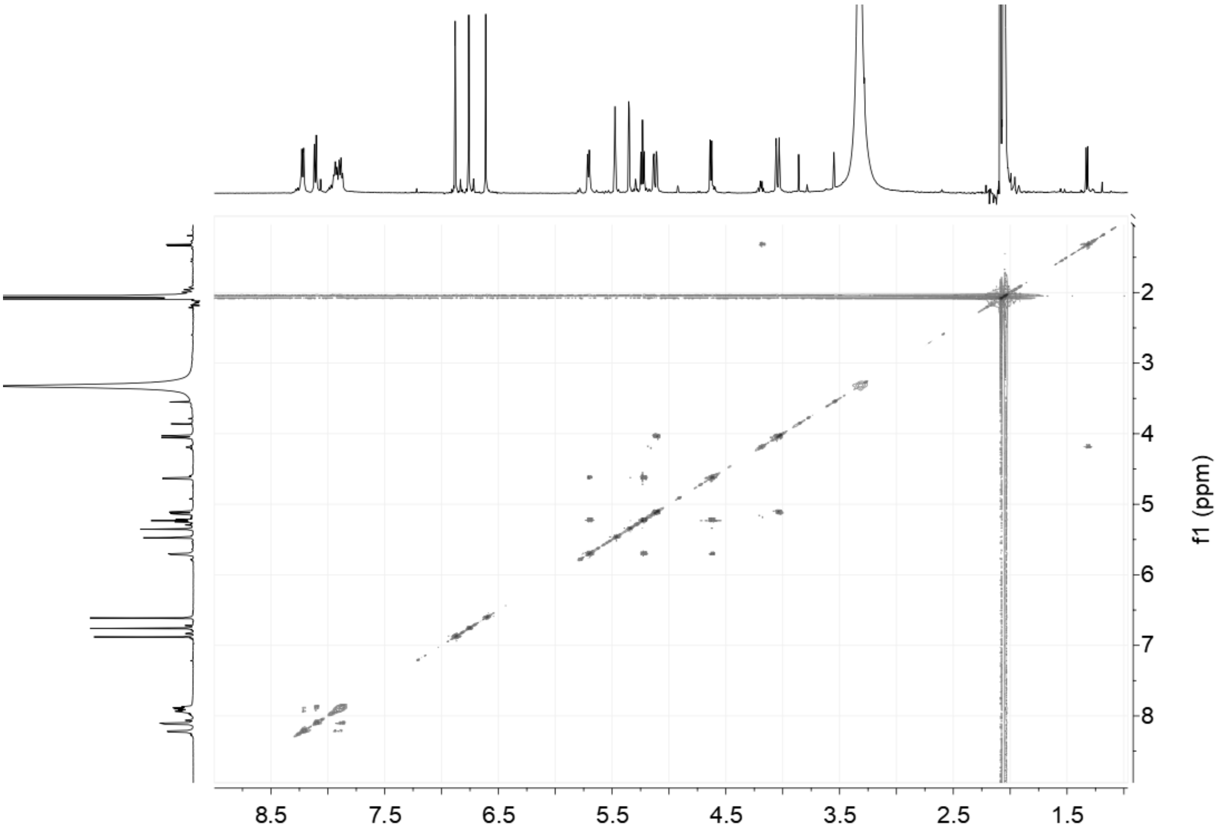

Figure S7. HR-FAB-MS spectrum of **6**

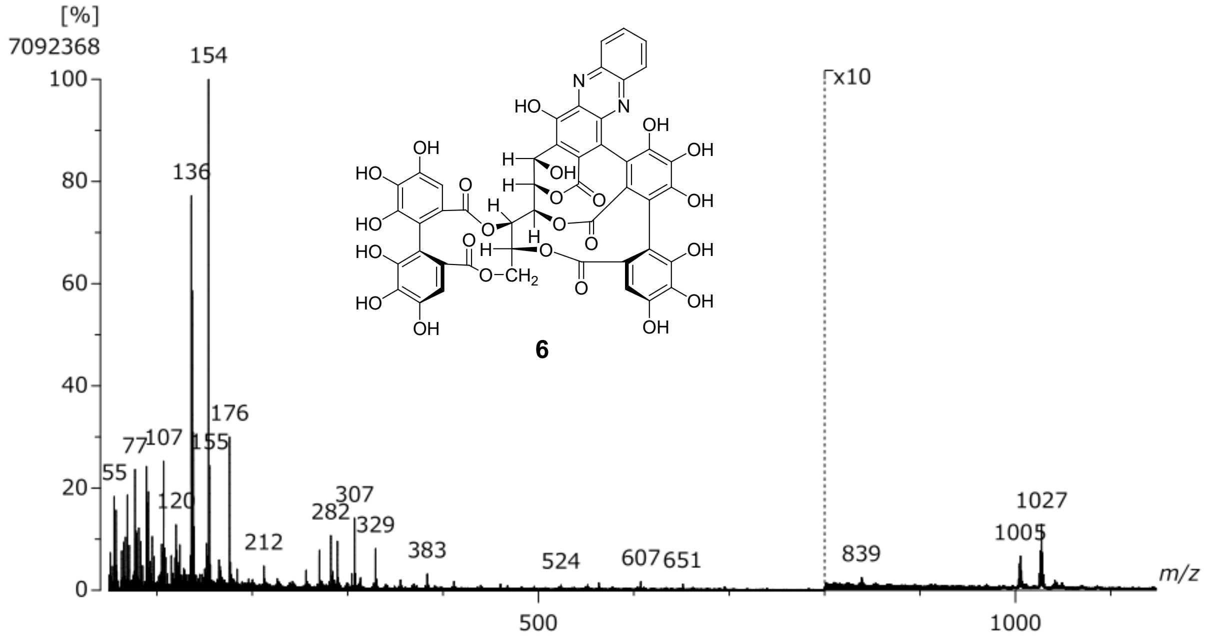

Observed m/z	Int%	Err[ppm / mmu]	U.S.	Composition
1027.0937	100.00	+0.7 / +0.7	34.5	C47 H28 N2 O24 Na

Figure S8. ^{1}H NMR spectrum of **7** in acetone-d_6.

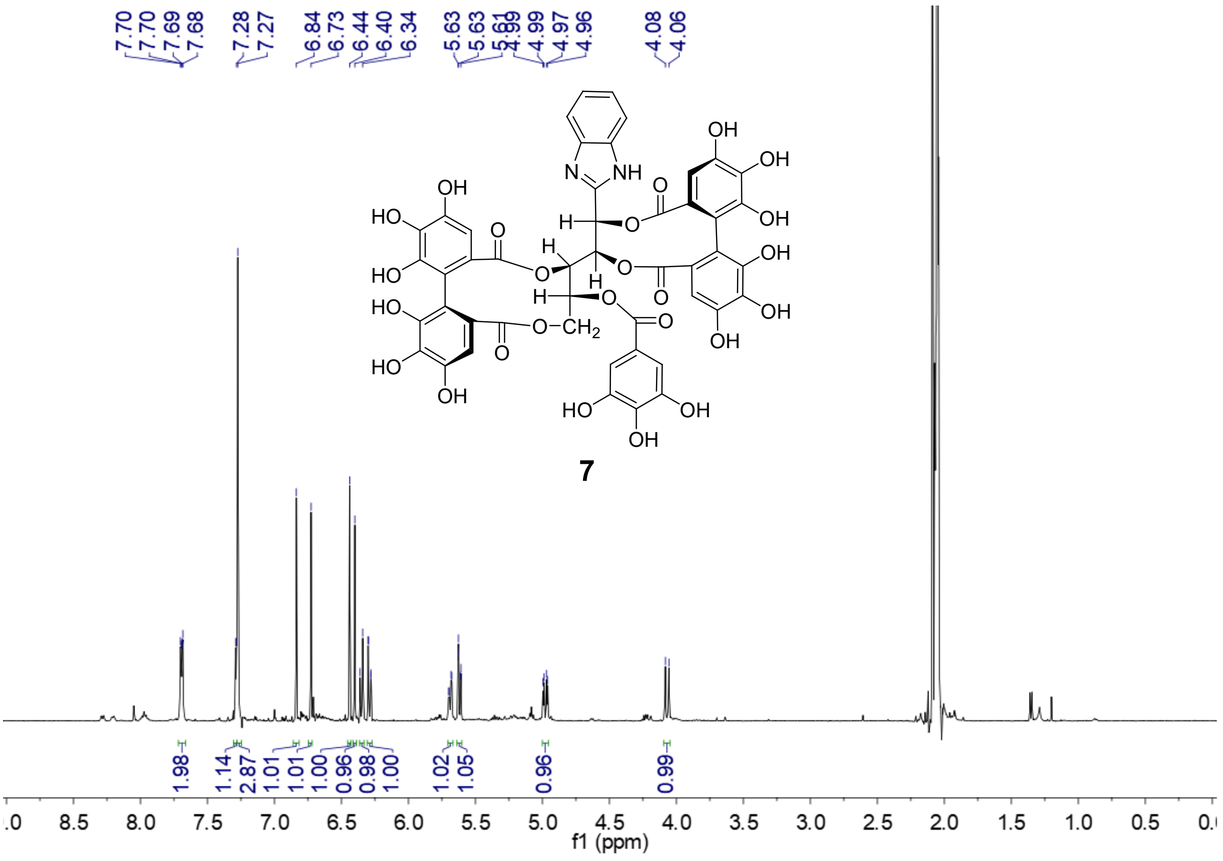

Figure S9. ^{13}C NMR spectrum of **7** in acetone-*d*6.

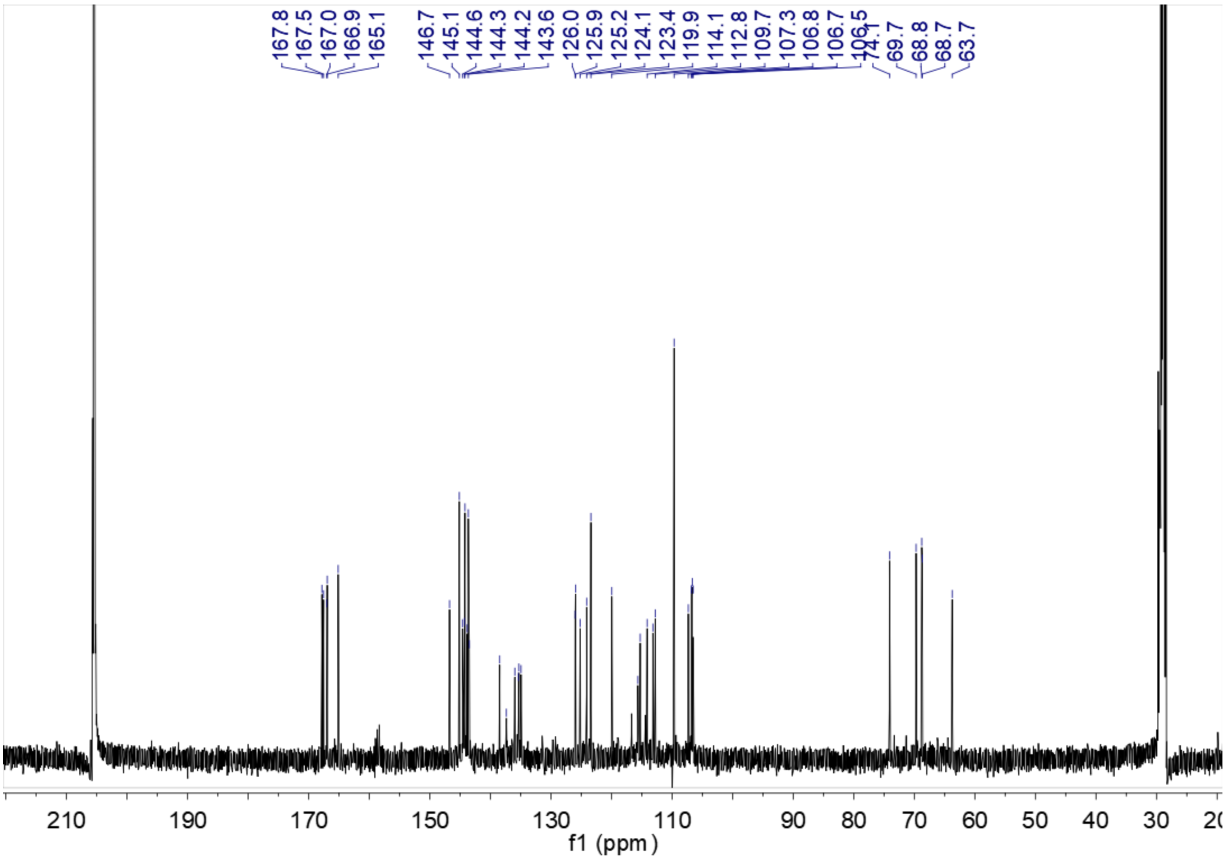

Figure S10. ^{1}H-^{1}H COSY spectrum of **7** in acetone-*d*6.

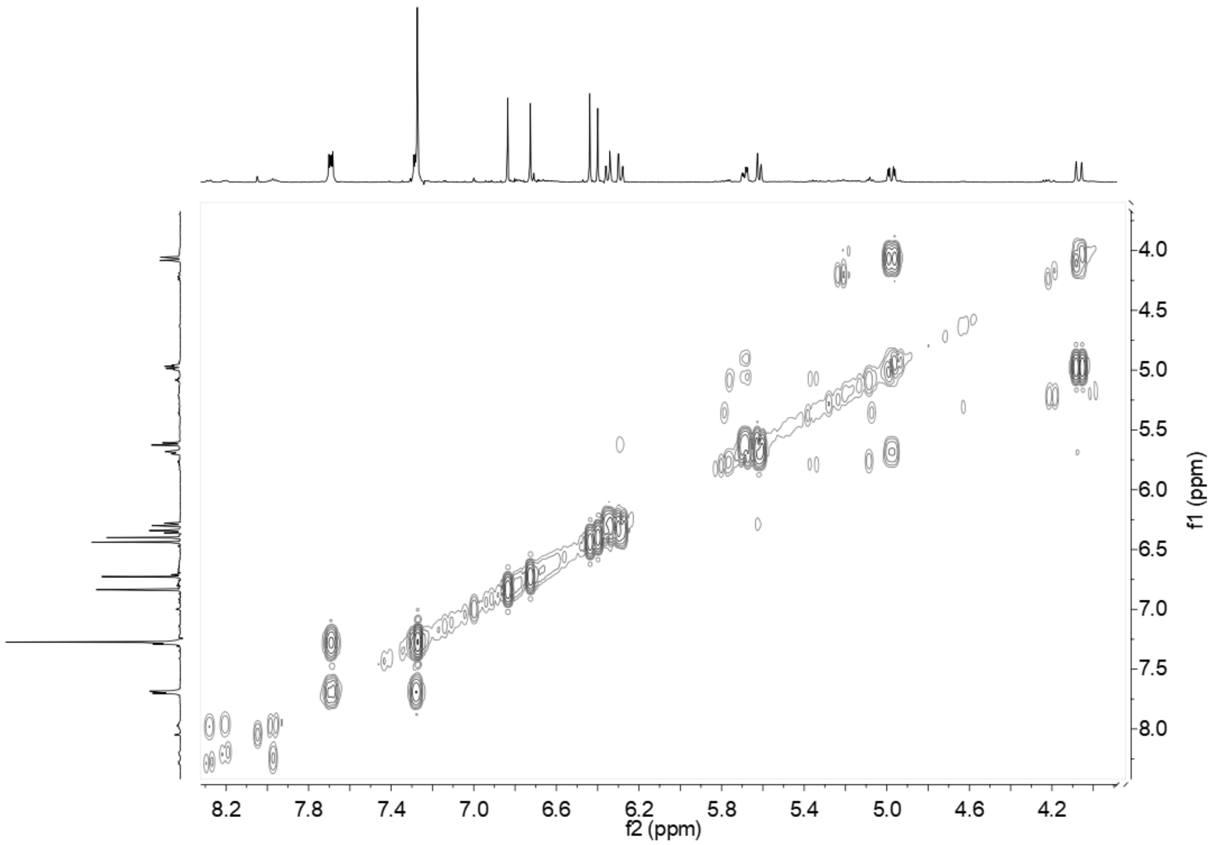

Figure S11. HSQC spectrum of **7** in acetone-d_6.

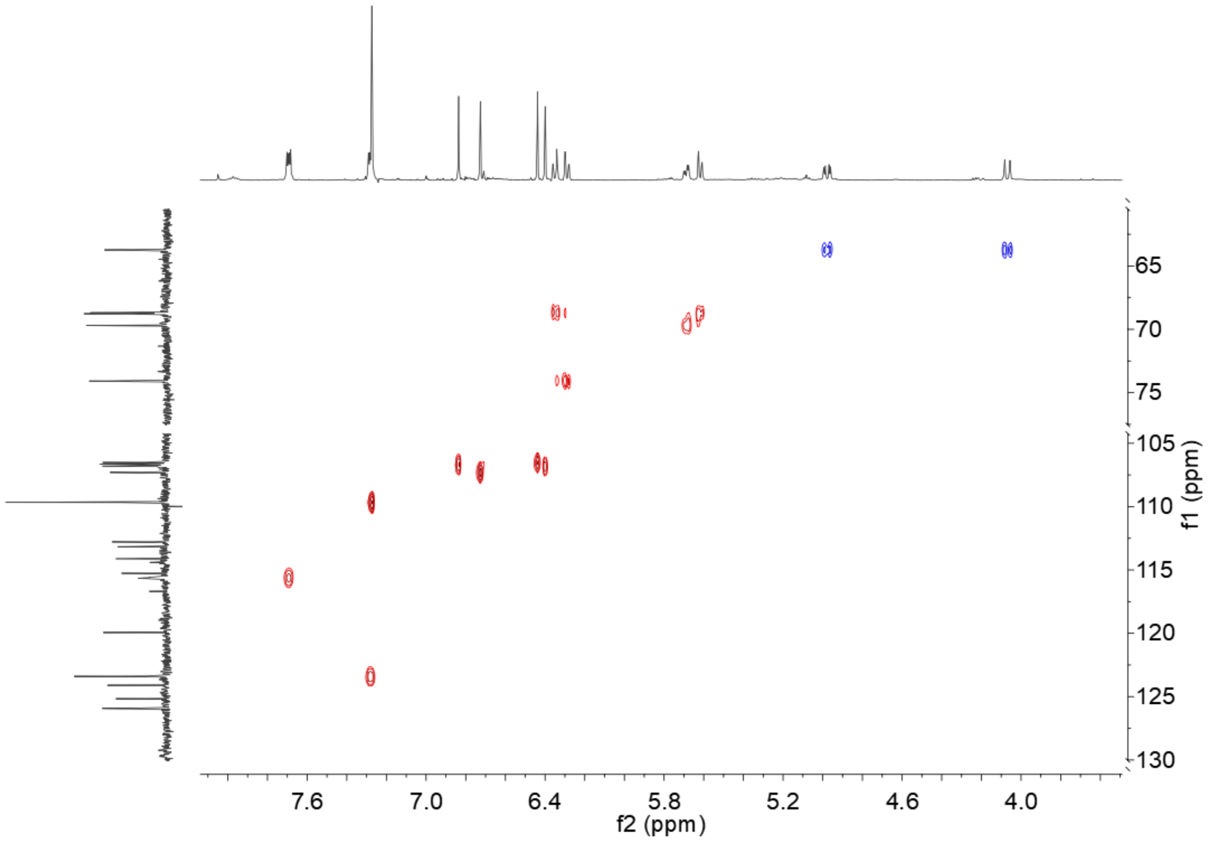

Figure S12. HMBC spectrum of **7** in acetone-d_6 (measured at 10Hz).

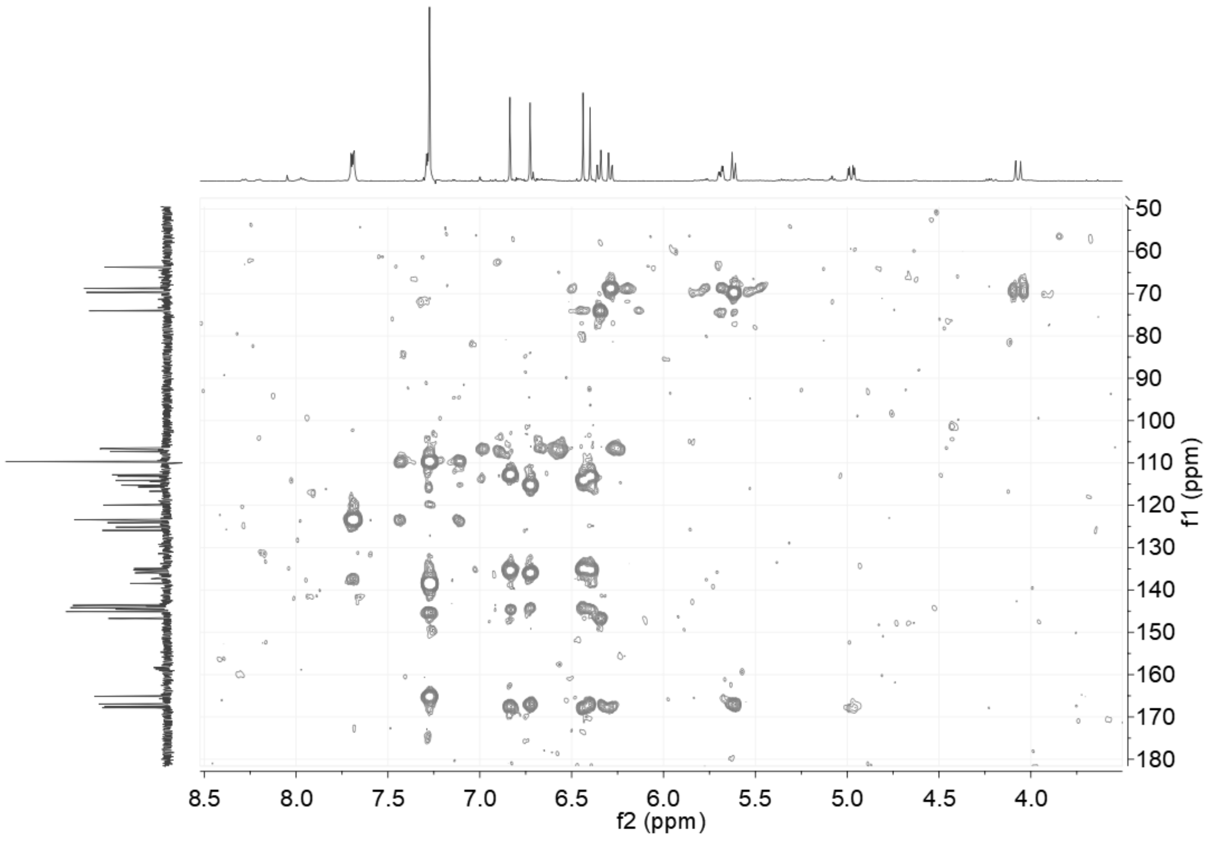

Figure S13. HMBC spectrum of **7** in acetone-d_6 (measured at 5Hz) .

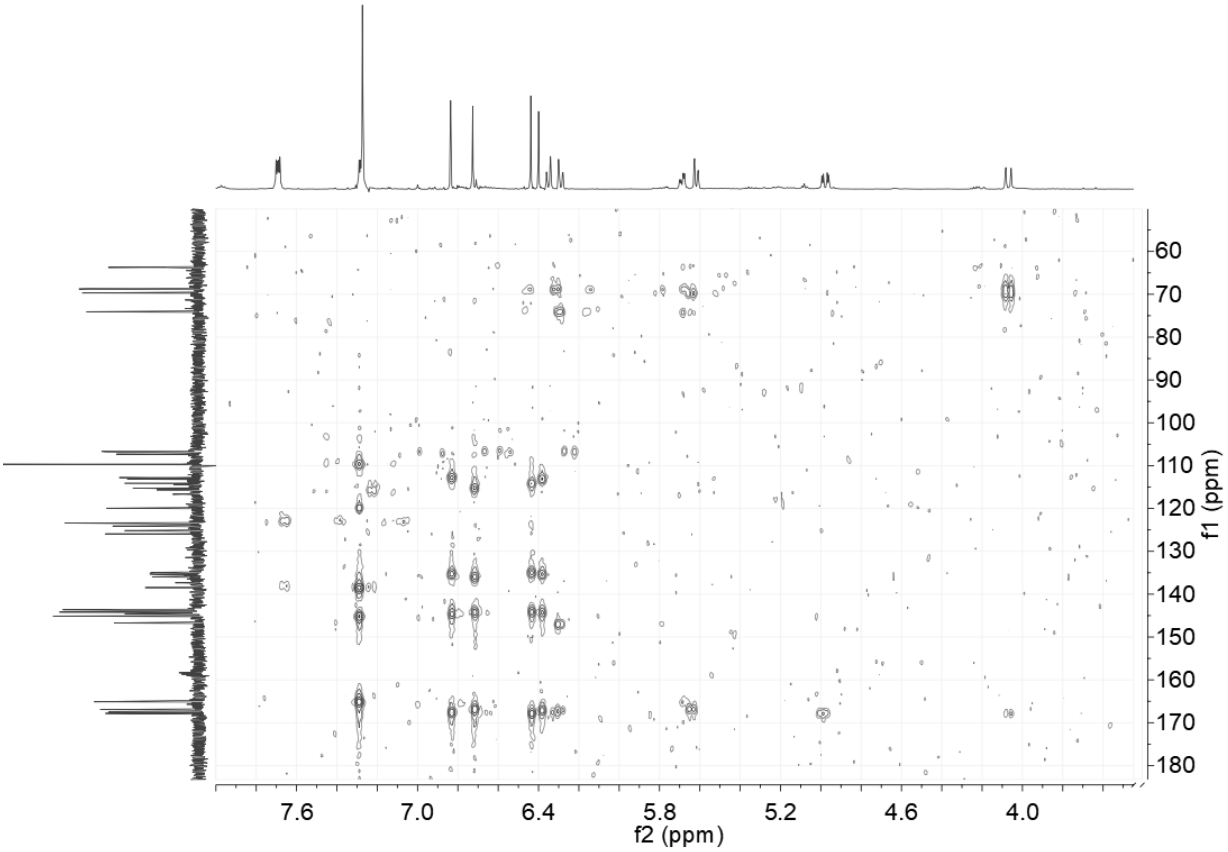

Figure S14. NOESY spectrum of **7** in acetone-d_6.

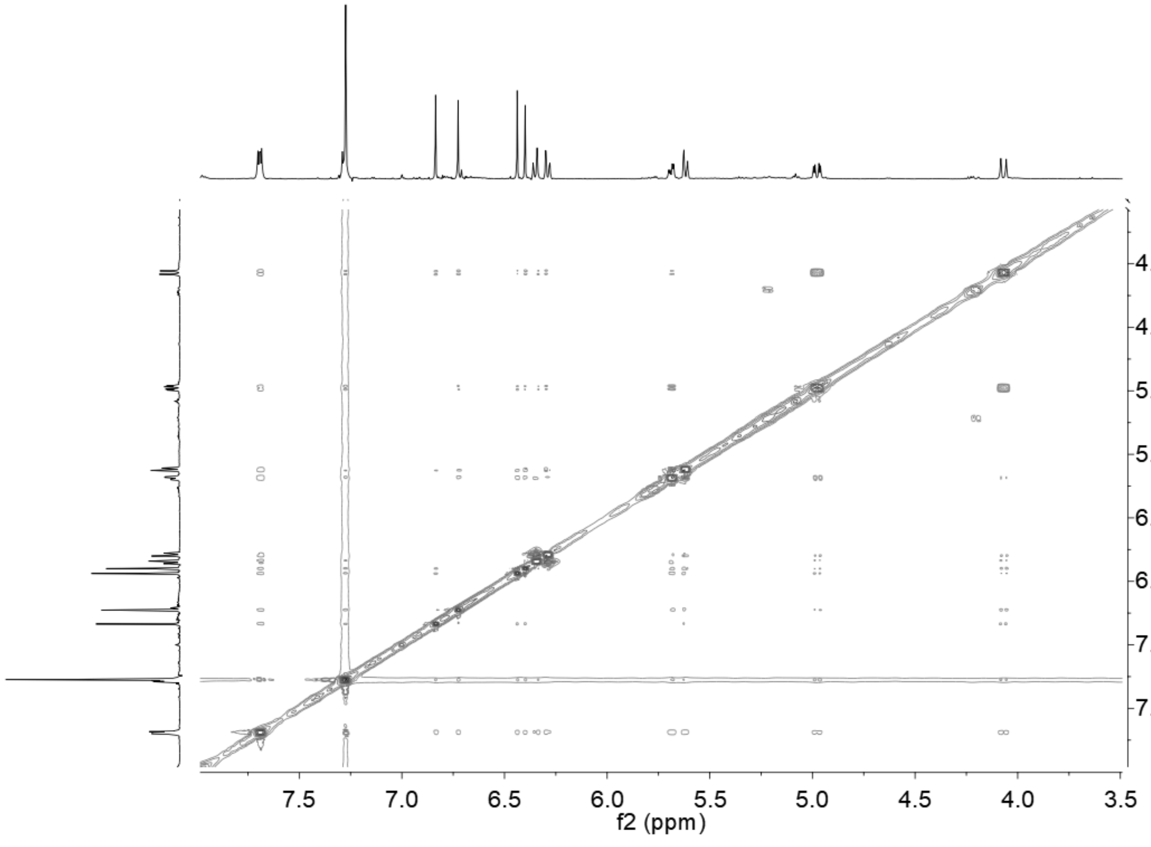

Figure S15. HR-FAB-MS spectrum of **7.**

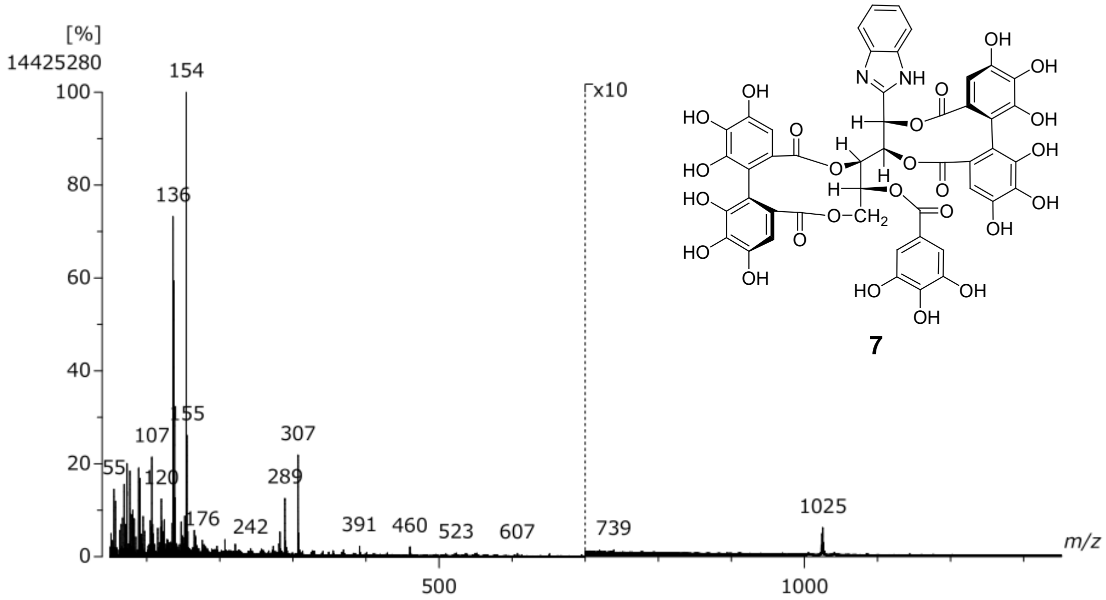

Observed m/z	Int%	Err[ppm / mmu]	U.S.	Composition
1025.1371	100.00	-0.1 / -0.1	32.5	C47 H33 N2 O25

Figure S16. ^{1}H NMR spectrum of **8** in acetone-d_6.

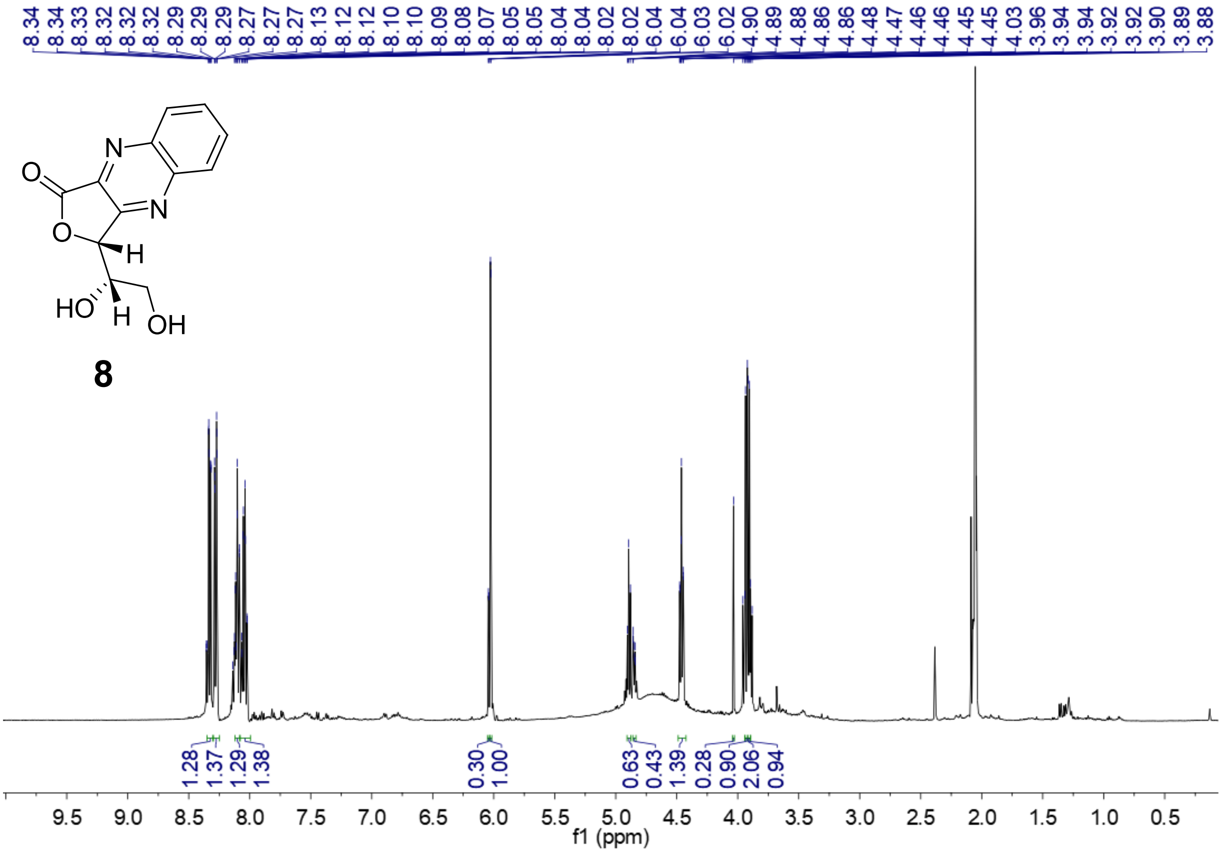

Figure S17. ^{13}C NMR spectrum of **8** in acetone-d_6.

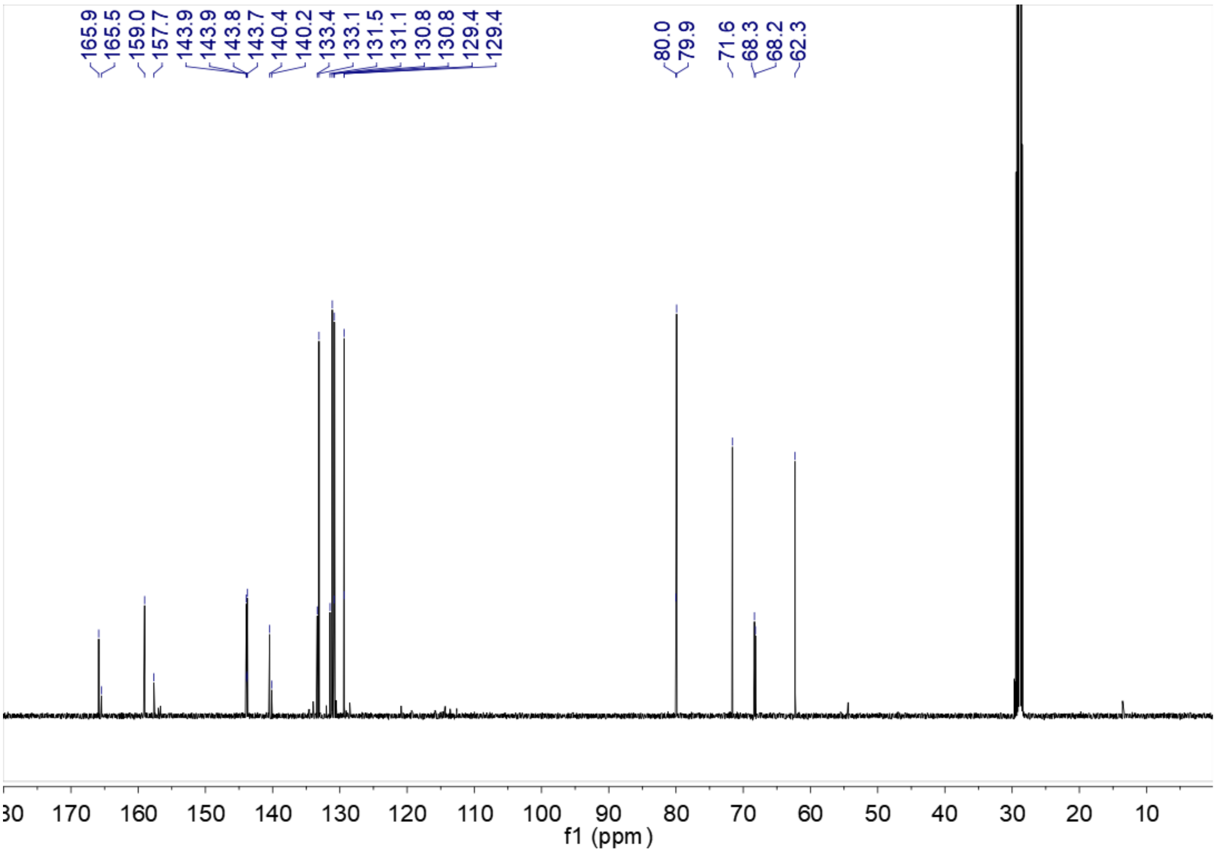

Figure S18. ^{1}H-^{1}H COSY spectrum of **8** in acetone-d_6.

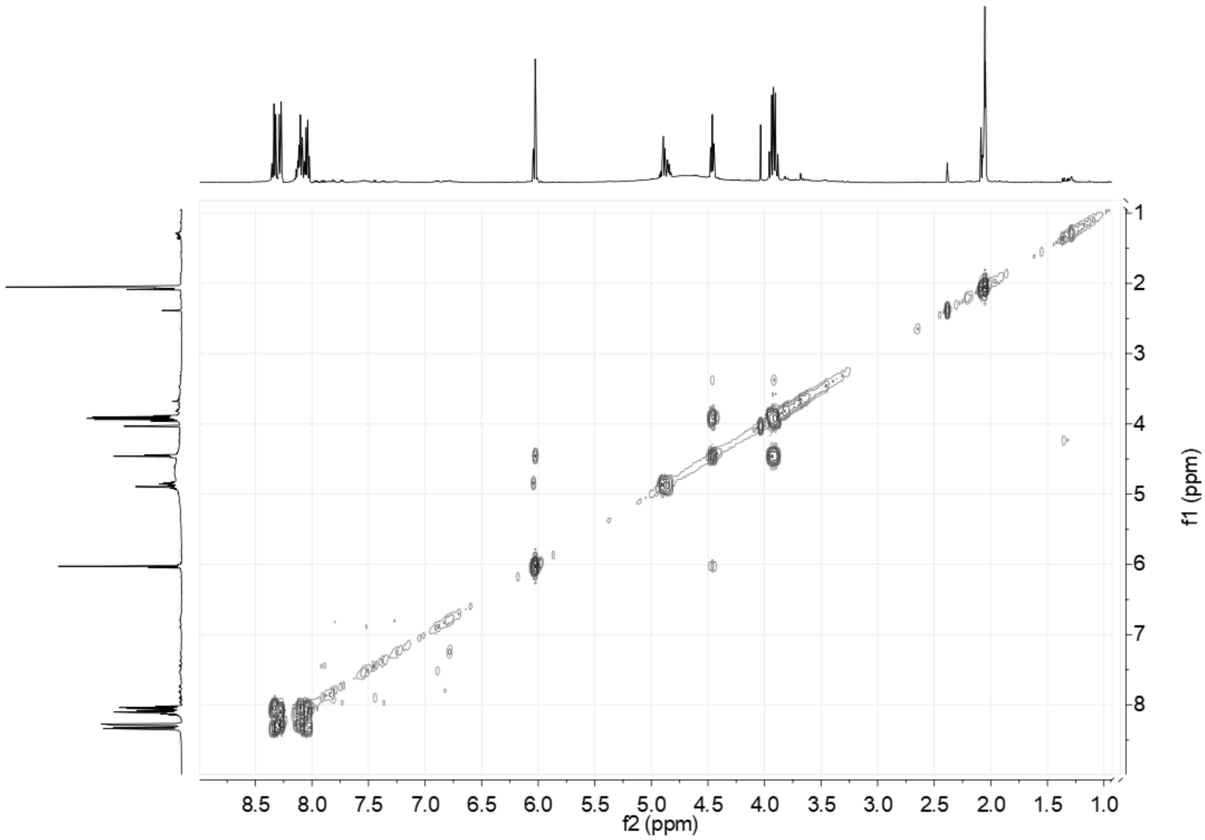

Figure S19. HSQC spectrum of **8** in acetone-d_6.

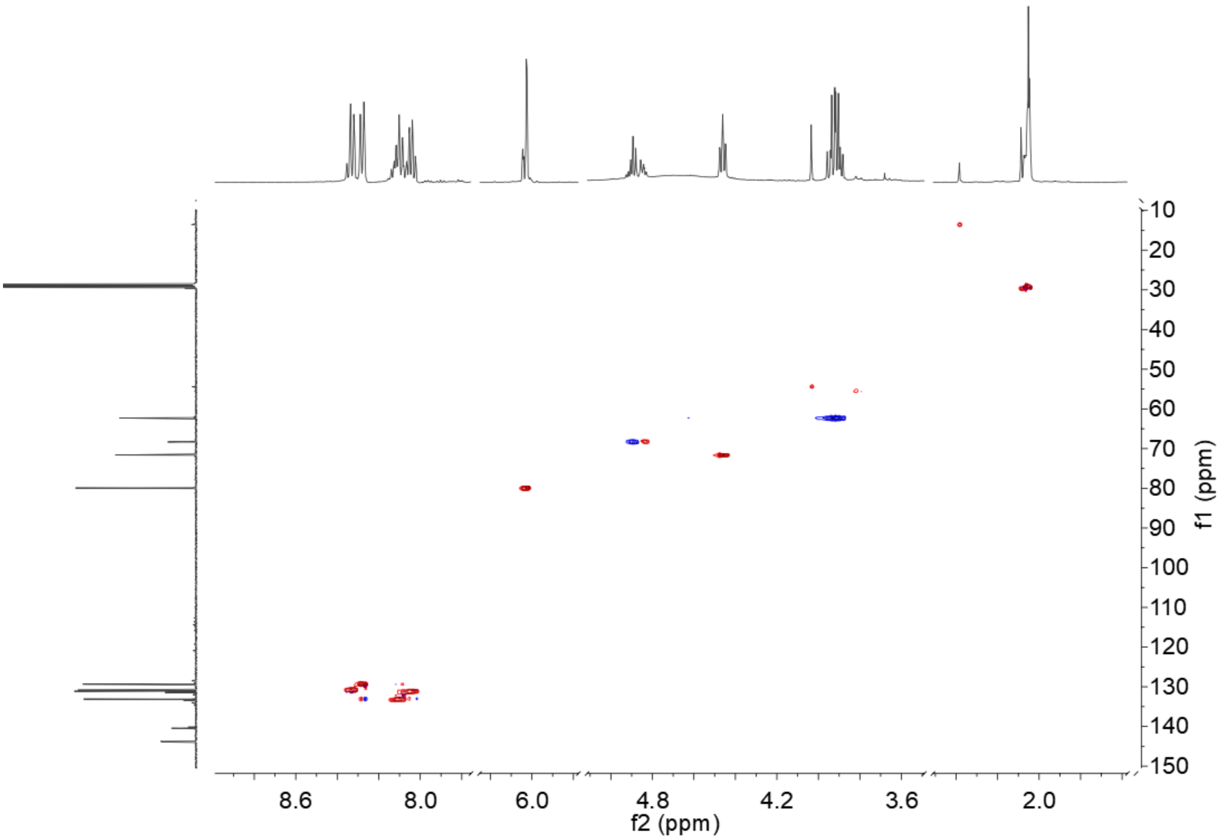

Figure S20. HMBC spectrum of **8** in acetone-d_6

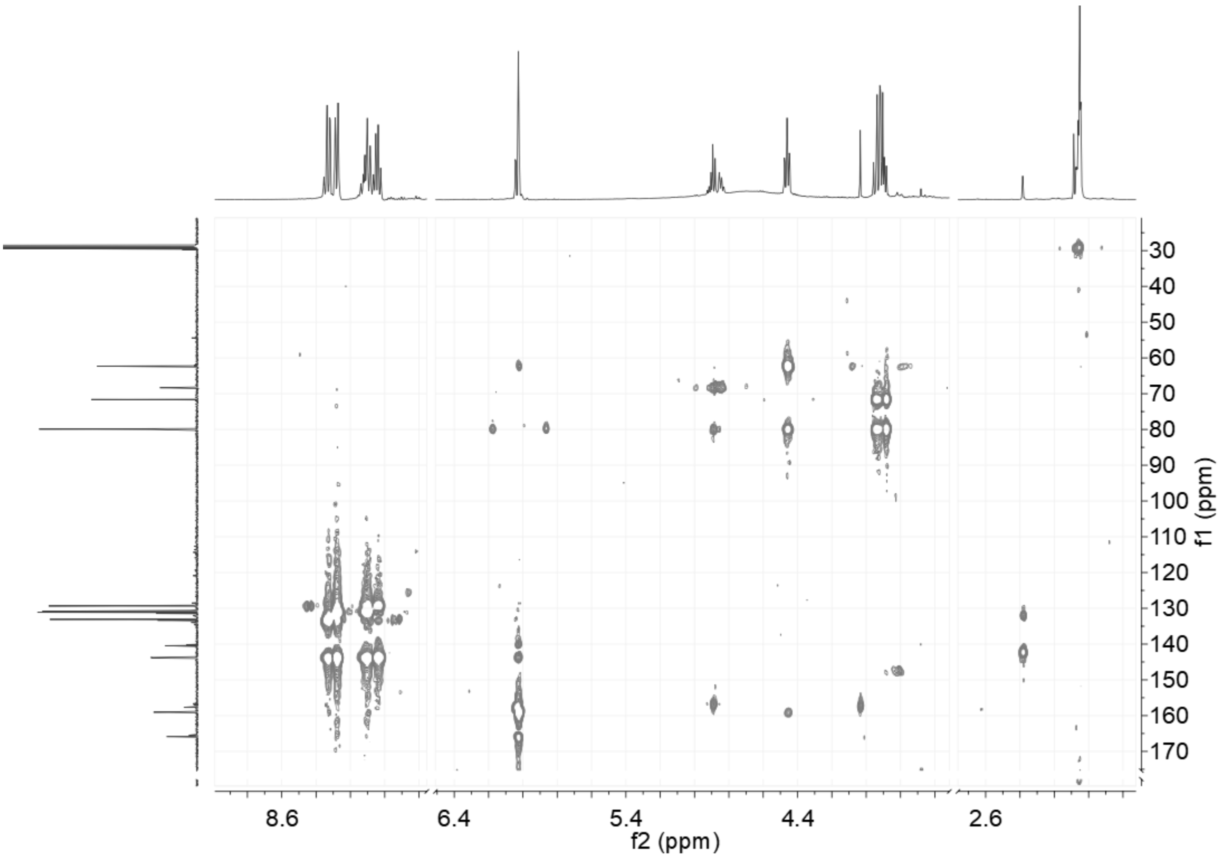

Figure S21. Roesy spectrum of **8** in acetone-d_6

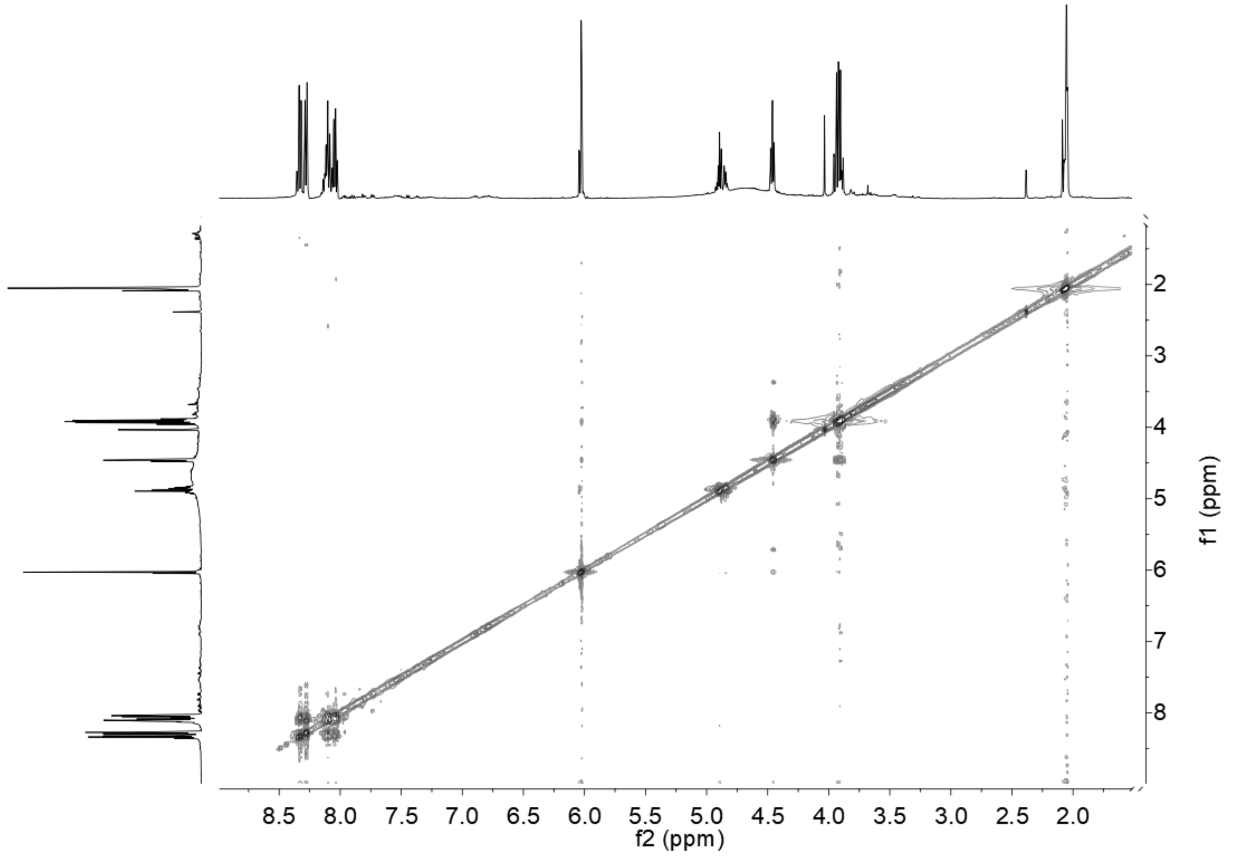

Figure S22. FAB-MS spectrum of **8**

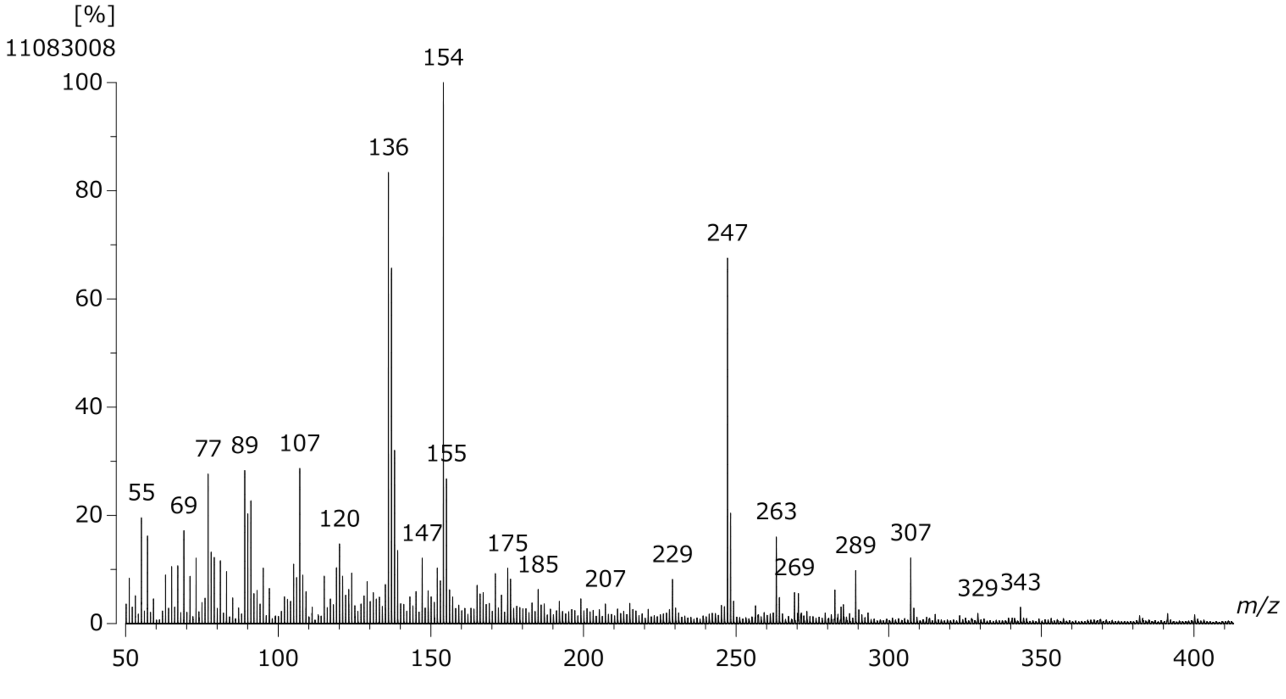

Supplement: Supplementary file 1 [file molecules-28-01246-s001.zip › molecules-2184889-supplementary.pdf]
